# Effects of Intercalation on ML-Ti_3_C_2_T_*z*_ MXene Properties and Friction Performance

**DOI:** 10.1021/acsami.4c12659

**Published:** 2024-11-06

**Authors:** Kailash Arole, Savannah E. Pas, Ratul Mitra Thakur, Lara A. Amiouny, M. Humaun Kabir, Milos Dujovic, Miladin Radovic, Jodie L. Lutkenhaus, Micah J. Green, Hong Liang

**Affiliations:** † Department of Materials Science and Engineering, Texas A&M University, College Station, Texas 77843, United States; ‡ Artie McFerrin Department of Chemical Engineering, Texas A&M University, College Station, Texas 77843, United States; § J. Mike Walker ’66 Department of Mechanical Engineering, Texas A&M University, College Station, Texas 778843, United States

**Keywords:** 2D nanomaterials, MXenes, interlayer spacing, friction, electrical conductivity

## Abstract

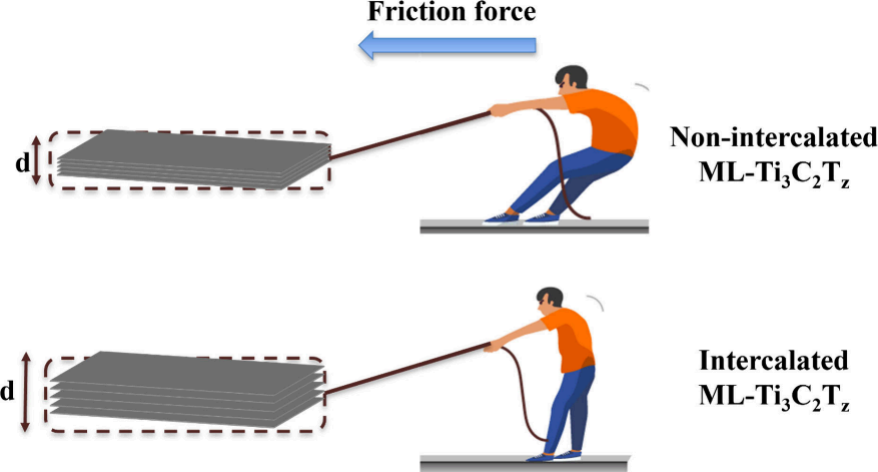

Intercalation in two-dimensional (2D) materials can modify their physical, chemical, and electronic properties. This modification enables the tailoring of 2D material characteristics, enhancing their performance and expanding their applications in various fields. The friction performance of 2D materials such as MoS_2_ and graphite has a strong dependence on their interlayer spacing, and they exhibit an increase in *d*-spacing associated with a reduction in friction performance. The ability to control the interlayer spacing of Ti_3_C_2_T_*z*_ MXene has proven beneficial for energy storage applications such as batteries and supercapacitors, but no one has utilized this control of interlayer spacing for lubrication. In this study, we demonstrate that interlayer spacing of multilayer (ML) Ti_3_C_2_T_*z*_ MXene can be controlled through chemical intercalation and its direct effects on the electrical conductivity and friction performance. We observed a notable decrease in electrical conductivity in vacuum-filtered ML-Ti_3_C_2_T_*z*_ MXene films, which was attributed to an increased internal resistance resulting from the expansion of the interlayer gap. We also found a significant reduction in the coefficient of friction for ML-Ti_3_C_2_T_*z*_ MXene with an increased *d*-spacing. This reduction is attributed to a weakened attraction of individual ML-Ti_3_C_2_T_*z*_ MXene layers (intercalated). Under a tangential force, it becomes easier to slide within the larger interlayer gap with weakened van der Waals forces. This work provides insights into the tunability of MXene properties through interlayer spacing, offering potential applications requiring materials with specific electrical and friction characteristics.

## Introduction

Two-dimensional (2D) materials such as graphite, molybdenum disulfide (MoS_2_), hexagonal boron nitride (h-BN), and zirconium phosphate (ZrP) have received tremendous attention due to their morphology, lubricity, high surface area, and low interlayer strength.^1−6^ In energy applications, they show great potential in developing next-generation batteries and supercapacitors, offering higher capacities and faster charging.^7,8^ Recently, the layered structure of 2D materials has been beneficial for tribological applications to minimize friction and wear.^9,10^

Intercalation has been reported as a versatile tool for fabrication and property tuning in 2D materials. The modification of interlayer spacing in 2D materials has demonstrated a significant impact on their physical and chemical properties.^11−13^ The control of interlayer spacing by intercalation can facilitate easier movement of ions and molecules in 2D materials, enhancing their ion storage capacity. Also, the expansion of layers in 2D materials like graphite and MoS_2_ has shown the influence on their 2D band structure. From a mechanical perspective, changes in interlayer spacing have shown the impact on the flexibility and strength of the material, making it more adaptable for use in flexible electronics.^14,15^

The unique layered structure of 2D materials results in low shear strength between individual layers due to weak van der Waals forces.^9,16^ These relatively weak forces between the layers reduce the friction during the sliding of two surfaces.^17,18^ The ability to control the distance between the layers can be helpful in controlling interfacial interactions for a range of target applications. Chemical intercalation is one way to control the distance between layers (*d*-spacing).^13,19^ The introduction of intercalants of different sizes results in altered interlayer spacing, and the increase or decrease of spacing alters the bonding strength between layers.^11,12,20^ The increased interlayer spacing promotes weakened van der Waals forces, providing a tailored approach to control friction.^11,20^ Additionally, the high in-plane mechanical strength of 2D materials ensures their structural stability under high-stress conditions, a vital attribute for maintaining low friction in demanding environments.^9^

Several experimental and theoretical investigations have reported that chemical intercalation alters the interlayer friction of materials such as graphite, MoS_2_, and h-BN.^11,20−23^ These reports suggest that the friction performance of lamellar compounds is strongly associated with *d*-spacing and weak van der Waals interactions of the basal planes or layers.^19,20,24,25^ The intercalation process improves the ability of adjacent layers to slide past one another under the application of shear forces.^23^ Additionally, increased *d*-spacing might lead to a reduction in the actual area of contact at the nanoscale, further reducing friction.^20^

Since the discovery of MXene by Naguib et al. in 2011, MXenes have received considerable attention owing to their excellent properties and applications in various fields.^3,26,27^ MXenes are the transition metal carbides, nitrides, or carbonitrides with the general formula of M_*n*+1_X_*n*_T_*z*_ (*n* = 1 to 4), where M is an early transition metal (e.g., Ti, V, Nb), X can be carbon or/and nitrogen, and T_*z*_ stands for terminal groups such as −OH, =O, −F, −Cl, −Br, −I, and −S.^28−30^ MXenes are prepared by the selective etching of the “A” layer (group 13 to 16 elements such as Al, Si) from the precursor MAX phase (M_*n*+1_AX_*n*_ where *n* = 1 to 4).^31^ More than 50 types of MXenes have been reported, with more being computed or synthesized, considering the possible compositions and solid solutions.^32−34^

The conventional wet etching approach is used in the majority of MXene synthesis reports; this procedure uses concentrated hydrofluoric acid (HF) as an etchant.^35^ However, an alternative approach is combining hydrochloric acid (HCl) with fluoride salts such as lithium fluoride (LiF) ammonium bifluoride (NH_4_HF_2_).^36^ This approach (mixing of fluoride salts with HCl) minimizes some of the practical hazards associated with handling of concentrated HF acid directly.^37^ The synthesis of MXene can also be performed by methods such as electrochemical etching, hydrothermal etching, and molten salt etching.^38−42^ The method of synthesis and intercalation directly affects the morphology, surface functionality, and properties of MXenes.^19^

One of the key benefits of ML-Ti_3_C_2_Tz MXenes is their controllable electrical conductivity, which is highly dependent on the interlayer spacing.^19^ Intercalation of MXene can result in an alteration of the interlayer spacing, directly influencing the movement of charge carriers within the material. Larger interlayer spacing generally enhances ionic diffusion and reduces electron scattering, which can improve conductivity.^19^ This tunability makes MXenes highly versatile for applications requiring precise control over electrical properties, such as energy storage devices, sensors, and conductive coatings.^13,24,43^ Also, the control of interlayer spacing by intercalation can facilitate the movement of ions and molecules in 2D materials, enhancing their ion storage capacity.^44,45^ Gandla et al. reported the advantage of larger interlayer spacing of a Mo_2_Ti_2_C_3_ MXene electrode toward an excellent performance supercapacitor in a binary ionic liquid–organic electrolyte.^44^

Multilayered MXene has been used as a lubricant additive in paraffin, mineral oil, and other base oils to enhance friction and wear performance compared to additive-free base oils, and 0.8–1.0 wt % loading of MXene exhibited about 54% reduction and 9-fold wear volume reductions.^18,46,47^ The single to few-layer MXene has also been evaluated as a lubricant additive exhibiting performance similar to multilayered MXenes.^48^ MXene has also been tested as a solid lubricant coating, and the method of application of the coating is very crucial to achieve good friction performance.^17,49^ The impact of chemical composition on friction and mechanical properties has been thoroughly investigated by Yang et al. in their study.^50^ They demonstrated that adhesion, along with the complex interactions between substrate and terminal groups, can either enhance or impede friction performance based on the distribution of terminal groups on the MXene surface. Overall, many reports have utilized MXenes as lubricant additives and solid lubricant coatings, but no one has utilized this control of interlayer spacing for lubrication.

In this work, we control the interlayer spacing between the ML-Ti_3_C_2_T_*z*_ MXene layers via chemical intercalation after etching. In order to alter the *d*-spacing, we investigated several different-sized intercalating agents such as lithium chloride (LiCl), sodium chloride (NaCl), urea (CH_4_N_2_O), dimethyl sulfoxide (DMSO), and tetrabutylammonium hydroxide (TBAOH). The change in the *d*-spacing of MXene alters the electrical conductivity and friction of ML-Ti_3_C_2_T_*z*_ MXenes. The increase in interlayer spacing resulted in a drop in the electrical conductivity of Ti_3_C_2_T_*z*_ films due to increased internal resistance. Additionally, the increased *d*-spacing or interlayer spacing of ML-Ti_3_C_2_T_*z*_ resulted in a reduction in the coefficient of friction. This decrease is attributed to the facilitated sliding of individual ML-Ti_3_C_2_T_*z*_ layers under applied shear forces or load, resulting from the weakened van der Waals interactions due to the increased interlayer spacing.

## Experimental Section

### Materials

The Ti_3_AlC_2_ MAX phase (Purity 99%) was prepared as previously reported.^40^ Lithium fluoride (LiF, 98%+) was purchased from Alfa. Hydrochloric acid (HCl, 37%, ACS reagent), dimethyl sulfoxide (DMSO, >99.5%), lithium chloride (LiCl), sodium hydroxide (NaOH), urea, and tetrabutyl ammonium hydroxide (TBAOH) were purchased from Sigma-Aldrich. All reagents were used as received without further purification.

## Methods and Characterization

### Preparation of ML-Ti_3_C_2_T_*z*_

Ti_3_C_2_T_*z*_ was obtained using our previously reported method. Briefly, 0.8 g of LiF was added to 10 mL of 6 M aqueous HCl solution with continuous stirring. Next, 1 g of Ti_3_AlC_2_ MAX powder was slowly added to this solution, and the mixture was stirred continuously at 40 °C for 40 h. The resulting suspension was washed with deionized water and separated by centrifugation, after which the supernatant was discarded. Moreover, this washing process was repeated several times until the supernatant reached pH ∼ 6. Then, the precipitate (ML-Ti_3_C_2_T_*z*_) was dispersed and intercalated.

### Intercalation of ML-Ti_3_C_2_T_*z*_

The conditions for the intercalation process were carefully controlled and followed the previously reported parameters.^25,51^ The etched ML-Ti_3_C_2_T_*z*_ was dispersed in the solution of LiCl, NaOH, urea, DMSO, and TBAOH. The reaction was performed at room temperature (∼25 °C) and lasted for 12 h with constant stirring, allowing sufficient time for effective intercalation. Excess intercalants were removed by centrifugation followed by water washing 3–4 times at 9000 rpm for 30 min, discarding the supernatant. The experiment was conducted under ambient atmospheric conditions, with no specialized gas environment required. Notably, the process was purely physical in nature, with no electrochemical potential or current applied. The intercalation of the chemicals was achieved through diffusion into the ML-Ti_3_C_2_T_*z*_ MXene layers, facilitated by prolonged stirring, which allowed for a thorough interaction between the chemicals and the MXene material. This straightforward method enabled efficient intercalation without the need for additional energy inputs.

### Solid Lubricant Coating

The obtained intercalated ML-Ti_3_C_2_T_*z*_ powder was dispersed in DI water to prepare a dispersion of 2 mg/mL. The resulting dispersion was subjected to bath sonication for 10 min and then drop cast onto a carbon-steel substrate, ensuring complete coverage of the substrate. The steel substrate was washed with a solution (50:50, % v/v) of acetone and isopropyl alcohol (IPA) in a bath sonicate for about 15 min. The drop-cast steel substrate was kept in a vacuum oven overnight to dry before testing.

### Scanning Electron Microscopy (SEM)

The FEI Quanta 600 field-emission scanning electron microscope was used to study the morphology of the Ti_3_C_2_T_*z*_. The supernatant was collected and freeze-dried to obtain a dry powder. SEM was performed on these dried powder samples using an acceleration voltage of 5–20 kV.

### X-ray Diffraction (XRD)

XRD was performed on freeze-dried samples (before and after annealed ML-Ti_3_C_2_T_*z*_) using a Bruker D8 powder X-ray diffractometer fitted with a LynxEye detector in a Bragg–Brentano geometry with a CuKα (l = 1.5418 Å) radiation source. A zero-background sample holder was used to test these samples with a scanning rate of 1.5 s per step and a step size of 0.02°.

### Optical Microscopy

Optical microscopy images were obtained by using an Amscope microscope. The samples were prepared by placing a drop of ML-Ti_3_C_2_T_*z*_ dispersion (in water) onto a glass slide.

### ζ Potential Measurement

Zetasizer Nano ZS90 from Malvern Instruments and DTS 1070 capillary cell from Malvern Instruments were used to determine the ζ potential of aqueous ML-Ti_3_C_2_T_*z*_ under ambient conditions. A diluted concentration of 0.05 mg/mL of the aqueous dispersion of ML-Ti_3_C_2_T_*z*_ was used to take the measurements.

### Four-Point Probe Conductivity

The electrical conductivity for each vacuum filtered film of ML-Ti_3_C_2_T_*z*_ was measured using the four-point probe method on a Resistivity Stand (S-302) procured from the Signatone Corporation.

### X-ray Photoelectron Spectroscopy (XPS)

An XPS/UPS system with an Argus detector was used for the XPS analysis of freeze-dried ML-Ti_3_C_2_T_*z*_. The sample holder was initially cleaned with isopropyl alcohol, and the samples were placed on the sample holder using conducting carbon tape. The high-resolution XPS spectra of all the elements were obtained using a pass energy of 20 eV and step size of 0.05 eV. The deconvolution spectra of Ti 2p, C 1s, O 1s, and Cl 2p were obtained using the procedure given by Halim et al.^52^ Component fitting was done using CasaXPS software, and a Shirley-type background function was used to determine the background contribution. The carbon peak (C–C, 284.8 eV) calibrated the component spectra. Elemental spectra were subsequently separated into the components for Ti_3_C_2_T_*z*_ and are listed separately in the graph. There were a few significant constraints while doing the peak fitting; first, all binding energies were allowed to shift in 0.02 eV intervals and were constrained to ±0.5 eV of their initial values. The full-width half-maximum values of components were also constrained. Finally, Ti components in the Ti 2p spectra were fit using asymmetric peaks with high binding energy tails due to their conducting behavior. All peaks and curves were fitted using Gaussian–Lorentzian curves.

### Profilometer

The thickness and roughness of ML-Ti_3_C_2_T_*z*_ coatings were evaluated using an Alpha-Step D-500 Stylus Profiler (KLA Instruments). The stylus used in this measurement was made of diamond. Each measurement was taken at three different locations of the coatings to minimize the error and obtain reliable data.

### Friction Measurements

The coefficient of friction was evaluated by using a tribometer with a pin-on-disk configuration. It consisted of a rotating disk (steel plate) and a fixed E52 100 steel bearing ball with a diameter of 6.35 mm. ML-Ti_3_C_2_T_*z*_ was dispersed in water (concentration 2 mg/mL) and bath sonicated for 15 min to obtain uniform dispersion, and about 0.2 mL of dispersion was drop cast on steel substrates (1 cm × 1 cm). The steel substrate was then vacuum-dried to form a solid lubricant film of ML-Ti_3_C_2_T_*z*_. The applied load was 1 N, and the sliding speed was 50 mm/s.

## Results and Discussion

**Figure 1:**
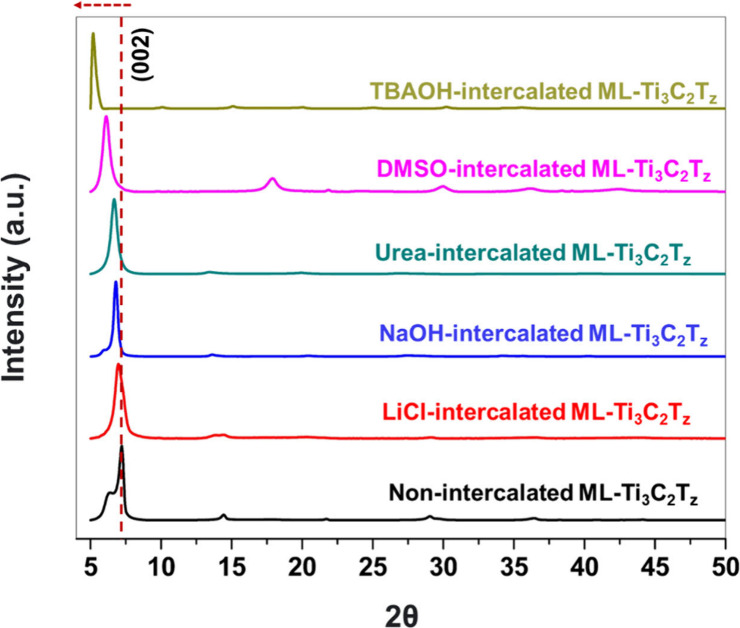
XRD of intercalated ML-Ti_3_C_2_T_Z_ MXene exhibiting a shift of the (002) peak to the left. (Note: the interlayer spacing (Å) calculated by Bragg’s law is included in the graphs).

The multilayered (ML) Ti_3_C_2_T_*z*_ MXenes used in this work are synthesized by acid etching (MILD method) as per the procedure reported earlier.^53^ The synthesized ML-Ti_3_C_2_T_*z*_ MXenes were further intercalated with different intercalating agents, which include lithium chloride (LiCl), sodium chloride (NaCl), urea (CH_4_N_2_O), dimethyl sulfoxide (DMSO), and tetrabutylammonium hydroxide (TBAOH). These intercalants were chosen based on their different sizes, as shown in Table S1. LiCl and NaCl are salts and when dissolved in water dissociate into their respective ions Li^+^ and Na^+^ which intercalate into ML-Ti_3_C_2_T_*z*_ MXenes,^13^ while on the other hand urea and DMSO intercalate as whole molecules.^25^ Also, TBAOH is an organic molecule, and when it is dissolved in water or another solvent, it dissociates into tetrabutylammonium cations (TBA^+^) to intercalate between MXene layers.^19,54^ These molecules will intercalate between the layers of ML-Ti_3_C_2_T_*z*_ MXenes and alter the interlayer spacing based on their sizes.^13,19,21,24^ X-ray diffraction (XRD) patterns of intercalated ML-Ti_3_C_2_T_*z*_ (Figure 1) were measured to understand the effect of intercalation on the structure of the MXenes. In the XRD analysis, no monochromator was used. The measurements were performed using standard X-ray optics, which allowed for sufficient resolution to analyze the interlayer spacing and crystal structure of the MXene samples. The (002) peak associated with the Ti_3_AlC_2_ MAX phase shifted from 2θ = 9.5° to 7.2° (Figure S1 and Figure 1), confirming the formation of ML-Ti_3_C_2_T_*z*_. The insertion or intercalation of ML-Ti_3_C_2_T_*z*_ with different intercalations (of varied sizes) resulted in the shift of the (002) peak to the left, indicating the increase of interlayer spacing. The highest shift in the (002) peak from 7.2° to 5.1° was observed for an ML-Ti_3_C_2_T_*z*_ intercalated with TBAOH due to the larger ionic size of the TBA^+^ ion.^55^ The absence of additional oxide peaks (22–26°) in the XRD of all intercalated samples suggests that the intercalation did not degrade the structure of ML-Ti_3_C_2_T_*z*_.

**Figure 2:**
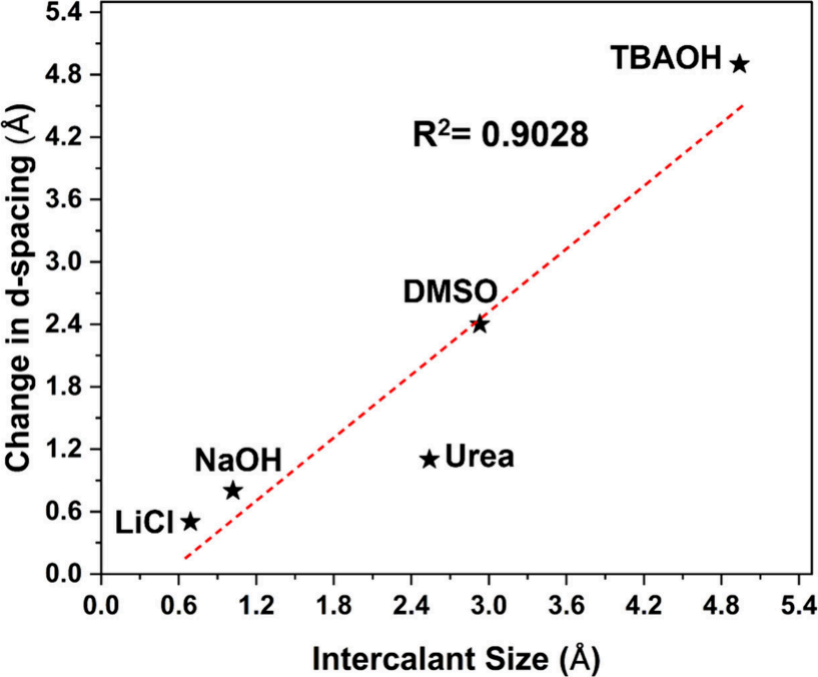
Linear fitting of the intercalant size and interlayer spacing in MXenes with an *R*^2^ value of 0.90 indicates the linear correlation between these two variables.

The (002) peak of the XRD of ML-Ti_3_C_2_T_*z*_ signifies the interlayer or *d*-spacing, which can be calculated using Bragg’s law.^13^Table 1 outlines the calculated *d*-spacing of ML-Ti_3_C_2_T_*z*_ MXenes intercalated with different intercalating agents. In this study, we focused on analyzing the impact of intercalation on the (002) diffraction peak to assess changes in the interlayer spacing. While other works have explored the detailed effects on the (040) and (060) peaks, as well as performed Rietveld refinement, these aspects were beyond the scope of our current investigation, and more details can be found in the literature.^13,19,24,25,56^Table S1 and Table 1 indicate that the *d*-spacing of ML-Ti_3_C_2_T_*z*_ MXenes increases as the size of intercalating agents increases. The intercalant sizes reported in Table S1 and Figure 2 represent the ionic radii of the respective ions, as commonly referenced in the MXene research community.^13,19,24,25,43,56^ The nonintercalated ML-Ti_3_C_2_T_*z*_ showed a *d*-spacing of 12.2 Å, which indicates the baseline spacing after etching.^57^ The highest increase in the interlayer spacing or *d*-spacing of ML-Ti_3_C_2_T_*z*_ was observed from 12.2 to 17.2 Å for the TBAOH-intercalated ML-Ti_3_C_2_T_*z*_. The observed increase in the spacing of MXene after the intercalation by a respective intercalant is in good agreement with the literature.^13,19,24,25,56^ The ability to control the *d*-spacing of MXene will be beneficial for lubrication performance.

**Table 1:** *d*-Spacing of Intercalated ML-Ti_3_C_2_T_*z*_ MXenes Calculated from the (002) Peak of the XRD Graph (Figure 1)

Sample name	*d*-spacing (Å)
Nonintercalated ML-Ti_3_C_2_T_*z*_	12.2
LiCl-intercalated ML-Ti_3_C_2_T_*z*_	12.7
NaOH-intercalated ML-Ti_3_C_2_T_*z*_	13.0
Urea-intercalated ML-Ti_3_C_2_T_*z*_	13.2
DMSO-intercalated ML-Ti_3_C_2_T_*z*_	14.5
TBAOH-intercalated ML-Ti_3_C_2_T_*z*_	17.2

Figure 2 illustrates the relationship between the size of intercalants and the resulting change in the *d*-spacing of ML-Ti_3_C_2_T_*z*_. Despite the relatively large size of urea, the change in the interlayer distance after intercalation is relatively small owing to the parallel orientation of urea molecules relative to the MXene surface as reported by Al-Temimy et al.^58^ The linear fit was applied on the obtained data, which is indicated with the red dashed line. The linear fitting with *R*^2^ = 0.9028 indicated the strong relationship between the intercalant size and interlayer spacing in MXenes. The prediction of interlayer distances is well-established for smaller ions with spherical configurations (e.g., Na^+^, Li^+^), and the intercalation of larger molecules such as tetrabutylammonium (TBA) introduces significant complexity.^54^ Multiple electrostatic forces, including hydrogen bonding, dipolar interactions, ionic forces, and van der Waals interactions, contribute to the molecular configuration within the rigid interlayer space.^13,24,25^ These forces result in nontrivial packing configurations, making precise predictions of the interlayer distance for large molecules challenging.^13,25^ This implies that the interlayer spacing in ML-Ti_3_C_2_T_*z*_ can be accurately predicted based on the intercalant size for designing MXene properties for various applications by selecting the appropriate intercalants.

**Figure 3:**
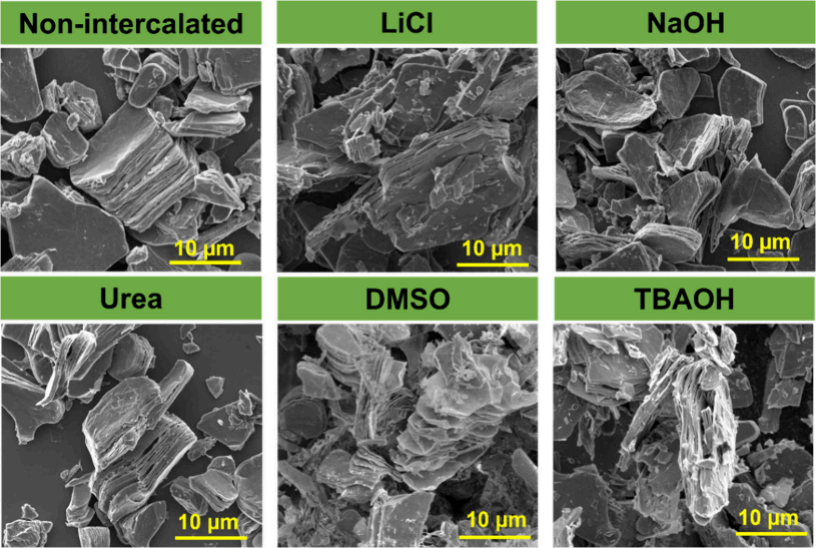
SEM of intercalated ML-Ti_3_C_2_T_Z_ MXene showing the expansion of the layers with the increased size of the intercalating agents.

The morphology of intercalated ML-Ti_3_C_2_T_*z*_ was further investigated by scanning electron microscopy (SEM), as shown in Figure 3. To minimize the possibility of cointercalated water molecules affecting the SEM imaging process, the samples were carefully dried before analysis. This procedure was aimed at reducing the presence of residual water that could evaporate under the vacuum conditions and heat of the SEM. The morphology of etched ML-Ti_3_C_2_T_*z*_ presents a typical accordion-like or layered structure. As-synthesized, nonintercalated MXene shows a slight expansion between the layers of ML-Ti_3_C_2_T_*z*_, with an interlayer spacing of 12.2 Å. However, after intercalating the multilayer MXenes, we observed increasing expansion between the layers of ML-Ti_3_C_2_T_*z*_ with increasing size of the intercalating agents (Table 1). While the SEM images (Figure 3) in this work were used to understand the effect of intercalation on morphology and microstructure, they are not intended for directly measuring interlayer spacing, particularly for materials like ML-Ti_3_C_2_T_*z*_ MXene where interlayer changes occur on the nanometer scale. Instead, the XRD analysis (Figure 1) discussed earlier was employed to accurately determine the interlayer spacing, as it is more suitable for assessing the structural changes in ML-Ti_3_C_2_T_*z*_ MXenes at the atomic level.

**Figure 4:**
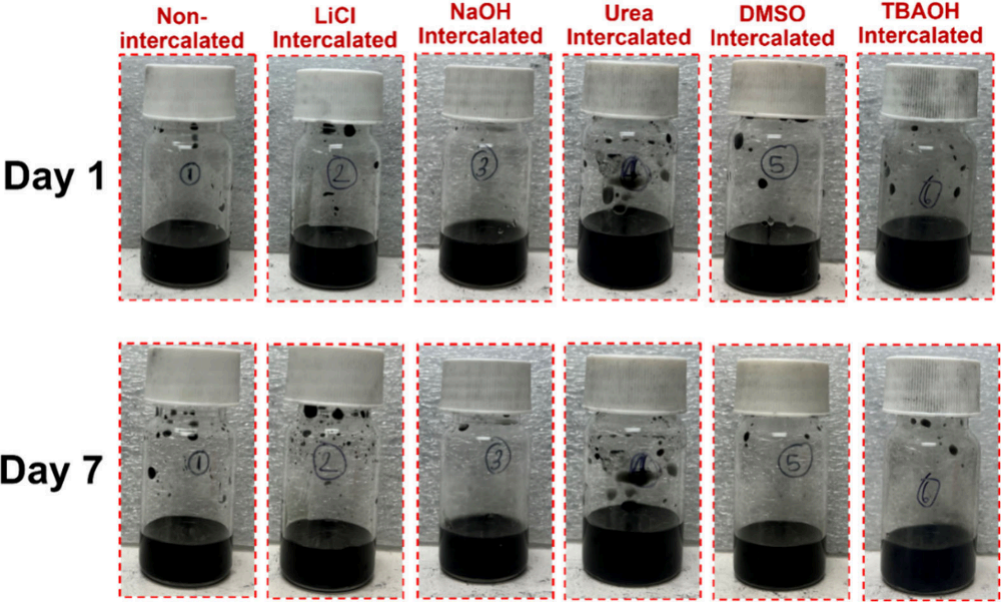
Images of an aqueous dispersion of ML-Ti_3_C_2_T_Z_ MXene.

The colloidal stability of the aqueous dispersion of intercalated ML-Ti_3_C_2_T_*z*_ MXenes was evaluated qualitatively by visual observations, as shown in Figure 4. The 1 mg/mL dispersion of ML-Ti_3_C_2_T_*z*_ MXene (nonintercalated and intercalated) was prepared simply by dispersing the dried powder in water and bath, sonicating the dispersion for 5 min to attain uniform mixing. The colloidal solutions were stored at room temperature (RT) under ambient conditions, without purging with inert gas. This approach was chosen to assess their stability in a typical environment that would be encountered during practical use of MXenes. The dispersion stability was observed visually for 7 days, and we did not see sedimentation of ML-Ti_3_C_2_T_*z*_ particles.

**Figure 5:**
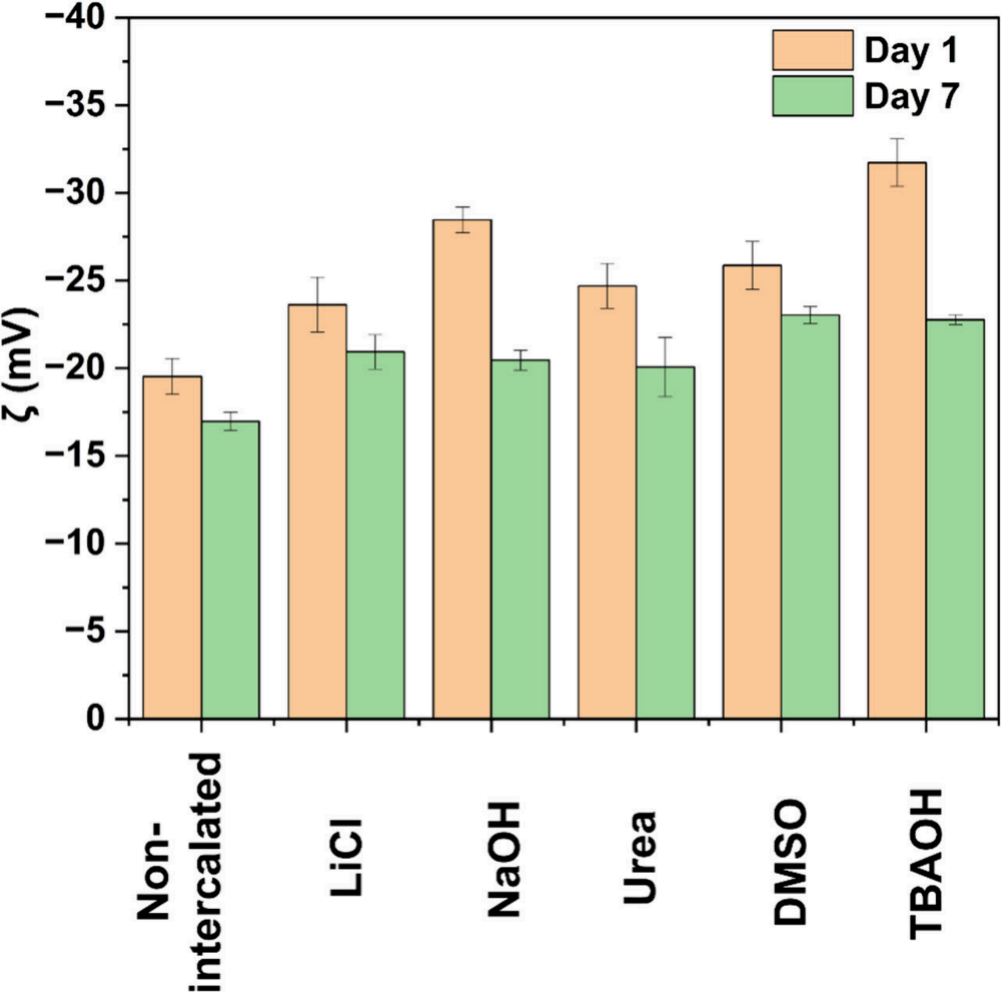
Zeta potential of aqueous dispersions of ML-Ti_3_C_2_T_Z_ MXene on day 1 and day 7.

Next, the stability of the aqueous dispersion of intercalated ML-Ti_3_C_2_T_*z*_ MXenes was investigated quantitatively by measuring their zeta potential (Figure 5). We ensured that all zeta potential measurements were performed under consistent conditions with the pH of the dispersions consistently maintained at approximately 6 for each sample. The negative zeta potential indicates strong repulsive forces between particles, keeping the ML-Ti_3_C_2_T_*z*_ MXenes separated and well-dispersed.^59^ The stronger zeta potential values in the case of NaOH and TBAOH can be attributed to the addition of hydroxyl groups (−OH) to the MXene surface during intercalation.^10,40^ It is important to note that the dispersibility is primarily determined by the magnitude of the zeta potential rather than its sign. While MXene dispersions commonly exhibit a negative zeta potential, the key factor for stability is the high absolute value, which promotes electrostatic repulsion and prevents particle agglomeration.^35^ The zeta potential measurements showed (Figure 5) a consistent decrease in surface charge from Day 1 to Day 7, indicating the dynamic changes in the colloidal stability over a time.

The dispersibility of ML-Ti_3_C_2_T_*z*_ MXenes was analyzed by optical microscopy imaging of aqueous dispersions. The dispersion quality plays a vital role in various applications such as thin film coatings and solid lubricants to attain uniform distribution of particles on the substrate.^10,60,61^ A well-dispersed lubricant reduces friction more effectively, leading to smoother operation of the coated surfaces.^17,62,63^Figure S2 shows that the intercalation of ML-Ti_3_C_2_T_*z*_ with different agents can lead to various morphologies and states of dispersion, which is significant for tailoring the properties of MXenes for specific applications. In this study, we focused on evaluating the dispersion state through optical microscopy, which provided sufficient insight into our specific research objectives (dispersion quality). Figure S2a is a control sample of ML-Ti_3_C_2_T_*z*_ without any intercalation treatment, and it shows stable dispersion without significant aggregation or sedimentation of particles. Also, the dispersion of intercalated ML-Ti_3_C_2_T_*z*_ MXenes exhibited a good distribution of particles in the optical images (Figure S2b–S2f), indicating good dispersibility. This suggests that intercalation of MXenes with different intercalating agents does not alter their dispersibility, at least in water. However, the slight difference in the images of urea intercalated ML-Ti_3_C_2_T_*z*_ compared to the others is due to the unique interaction between urea and the MXene surface, which can alter the aggregation state, particle size distribution, and overall dispersion stability, leading to distinct visual characteristics under an optical microscope. Overall, achieving good dispersion in solid lubricant coatings is essential for maximizing their effectiveness and durability. The intercalated and nonintercalated ML-Ti_3_C_2_T_*z*_ MXenes have shown a good dispersibility in hydrophilic solvents appropriate for coatings.^64−66^

**Figure 6:**
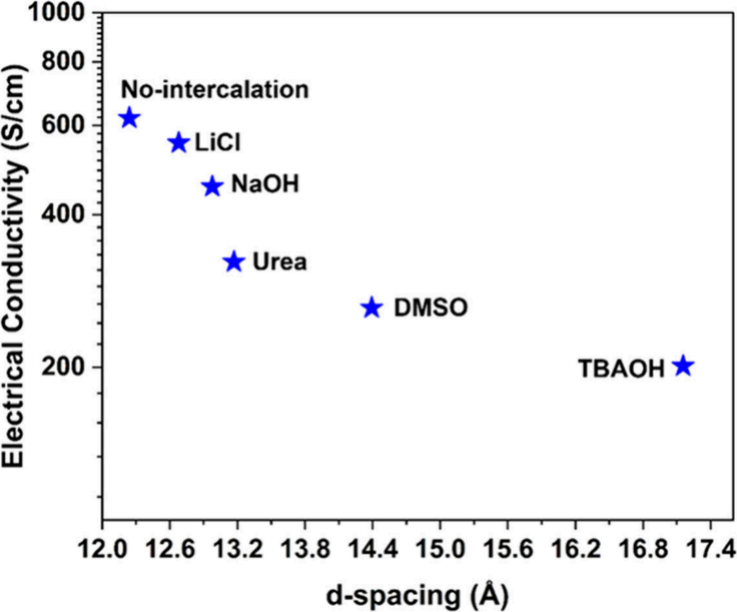
Effect of *d*-spacing on DC electrical conductivity of ML-Ti_3_C_2_T_Z_ MXenes intercalated with different intercalating agents.

MXene has been utilized in flexible wearable electronics due to the electrical conductivity of free-standing films.^67^ In this work, vacuum-filtered films were prepared to measure the electrical conductivity of nonintercalated and intercalated ML-Ti_3_C_2_T_*z*_ MXenes. The electrical conductivity for each vacuum-filtered film was measured by using the four-point probe method. Figure 6 outlines the relationship between the interlayer spacing or *d*-spacing and electrical conductivity for ML-Ti_3_C_2_T_*z*_ MXene with different intercalating agents. The “No-intercalation” case serves as a baseline or controlled sample, showing the electrical conductivity of the ML-Ti_3_C_2_T_*z*_ without any intercalation. In the case of intercalated MXene, we observed that with increased *d*-spacing the electrical conductivity of ML-Ti_3_C_2_T_*z*_ goes down, which is in agreement with earlier reports.^11,12,19−21^ This trend can be attributed to the increased interlayer resistance due to increased interlayer spacing.^19^ Increased *d*-spacing in ML-Ti_3_C_2_T_*z*_ can impede the flow of electrons between the layers of MXene, resulting in lower electrical conductivity.^19^ However, the substantial difference in electrical conductivity between NaOH- and urea-treated ML-Ti_3_C_2_Tz MXenes, despite having very close interlayer spacing, can be attributed to changes in surface chemistry and the unique interactions induced by each treatment, as evidenced by previous studies.^19,25^

**Figure 7:**
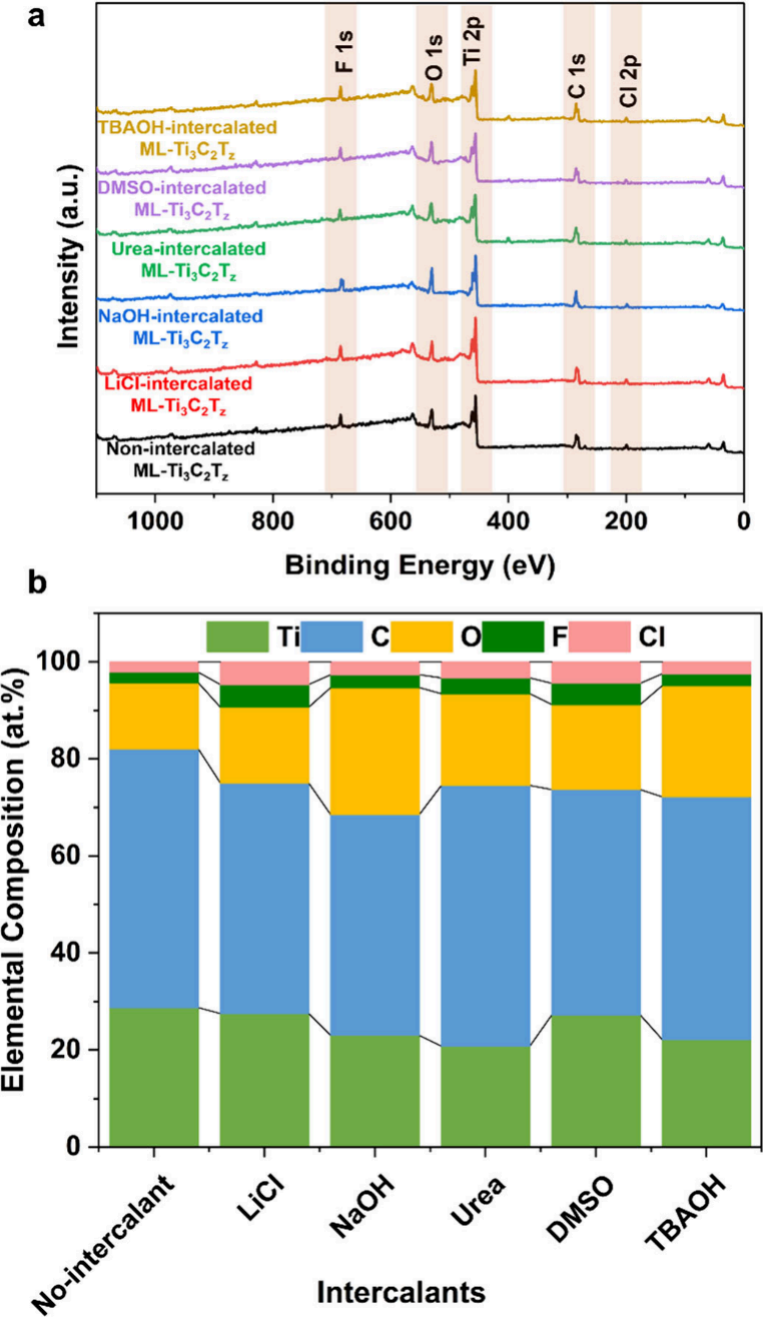
(a) XPS survey spectra and (b) corresponding elemental composition (at. %) of intercalated ML-Ti_3_C_2_T_*z*_ MXenes.

To gain further insights into the effect of intercalation on the terminal group distribution of MXene, X-ray photoelectron spectroscopy (XPS) was carried out on the vacuum-filtered films of nonintercalated and intercalated ML-Ti_3_C_2_T_*z*_. Figure 7a shows the survey spectra of ML-Ti_3_C_2_T_*z*_. The XPS spectra and deconvoluted high-resolution peak fits for the ML-Ti_3_C_2_T_Z_ MXenes can be found in the Supporting Information (Figures S3–S8). Figure 7b outlines the elemental composition (atom %) of nonintercalated and intercalated ML-Ti_3_C_2_T_*z*_, and the data indicate that all samples contain the characteristic elements Ti, C, O, F, and Cl. In all the cases, overall ML-Ti_3_C_2_T_*z*_, which are intercalated with NaOH and TBAOH, exhibited higher oxygen than the others, and that is due to the addition of hydroxyl groups on the ML-Ti_3_C_2_T_*z*_ surface.^40^ The deconvoluted Ti 2p spectra of all the ML-Ti_3_C_2_T_*z*_ MXenes (Figures S3–S8) showed the characteristic Ti–C (Ti–C 1/2) peak at 454.9 (460.9), which confirms the synthesis of Ti_3_C_2_T_*z*_.^52,68^ The at. % of Ti–C in all the samples were found to be in the range of 49–56% which is in agreement with the literature.^52^

In order to understand the presence of the OH group, we have deconvoluted the high-resolution O 1s spectra, and the C–Ti–OH content was calculated. C–Ti–OH refers to hydroxyl groups (−OH) that are chemically bound to the titanium (Ti) and carbon (C) atoms in the MXene structure. The C–Ti–OH content for nonintercalated ML-Ti_3_C_2_T_*z*_ was measured to be 22.75 at. %, while intercalation with different chemicals resulted in variations such as LiCl-intercalated (20.49 at. %), NaOH-intercalated (29.45 at. %), urea-intercalated (36.25 at. %), DMSO-intercalated (19.04 at. %), and TBAOH-intercalated (18.63 at. %). These variations indicate a correlation between the intercalation process and the concentration of OH functional groups present on the surfaces of MXenes. Also, the utilization of water during the washing of intercalant from MXene can also be put on the hydroxyl groups on MXenes.^40^ The C 1s region of as-made MXene and intercalated ML-Ti_3_C_2_T_*z*_ was fit by four components peaks, C–C, C–Ti–T_*z*_, CH/C–O, and COO, and the well-fitted components confirm the stability of MXene even after intercalation. This demonstrates that the structural integrity and chemical composition of the ML-Ti_3_C_2_T_*z*_ MXene are maintained, indicating successful intercalation without degradation of the material.

**Figure 8:**
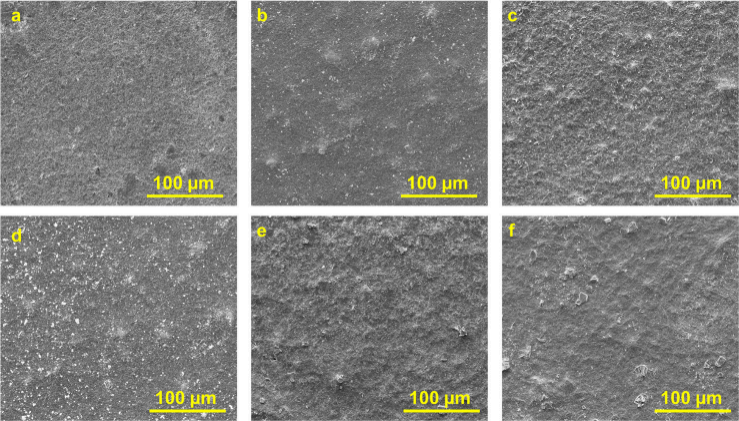
Surface morphology of ML-Ti_3_C_2_T_*z*_ MXene coated steel substrates intercalated with different intercalating agents: (a) no intercalant; (b) LiCl; (c) NaOH; (d) urea; (e) DMSO; and (f) TBAOH.

The solid lubricant coatings evaluated in this work are prepared from the fresh dispersions of ML-Ti_3_C_2_T_*z*_ (detailed procedure is mentioned in the Experimental Section) drop casting (Figure S9) on a steel substrate (Figure S11) and drying them under vacuum at room temperature (25 °C). The surface morphology of prepared solid lubricant coatings of ML-Ti_3_C_2_T_*z*_ MXene was investigated by SEM, as shown in Figure 8. It shows that the generated coating (Figure 8) was uniform with a very dense deposition of ML-Ti_3_C_2_T_*z*_, revealing a continuous and uniform material layer with minimal porosity or visible gaps. To further support the evidence of a homogeneous and dense structure, we included thickness measurements and surface roughness data in Table 2. These quantitative analyses, combined with the SEM images, provide a clearer understanding of the uniformity and density of the MXene-coated steel substrates. The ability of ML-Ti_3_C_2_T_*z*_ MXene to form a strong and uniform coating on a substrate is due to the presence of −OH terminal groups on ML-Ti_3_C_2_T_*z*_ (attached during etching), which has strong hydrogen bonding with the steel disk.^9,16^

**Table 2:** Profilometer (Thickness and Roughness Value) of Intercalated ML-Ti_3_C_2_T_*z*_ MXene-Coated Substrates

Sample name	Thickness (μm)	Roughness (μm)
Steel substrate (No coating)	-	0.15 ± 0.05
Nonintercalated ML-Ti_3_C_2_T_*z*_	5.87 ± 0.23	2.65 ± 0.80
LiCl-intercalated ML-Ti_3_C_2_T_*z*_	5.79 ± 0.20	2.44 ± 0.14
NaOH-intercalated ML-Ti_3_C_2_T_*z*_	6.95 ± 1.04	2.66 ± 0.44
Urea-intercalated ML-Ti_3_C_2_T_*z*_	6.70 ± 0.53	2.05 ± 0.19
DMSO-intercalated ML-Ti_3_C_2_T_*z*_	6.77 ± 0.58	2.23 ± 0.41
TBAOH-intercalated ML-Ti_3_C_2_T_*z*_	7.51 ± 1.05	2.32 ± 0.09

The thickness and roughness of solid lubricant coatings are critical parameters in determining their efficiency and durability.^48,63^ The measured thickness and roughness values of the ML-Ti_3_C_2_T_*z*_ coatings are outlined in Table 2. The steel substrate without any coatings used in this work showed a roughness of 0.15 ± 0.05 μm. The thickness values of nonintercalated and intercalated ML-Ti_3_C_2_T_*z*_ MXene coatings ranged from 5.87 to 7.51 μm, with low standard deviation. The higher surface roughness observed may be attributed to differential rates of etching, particularly due to the significant presence of F and Cl, as confirmed by XPS analysis, which can alter surface morphology. Although we did not perform elemental mapping in this study, the correlation between surface roughness and the high content of F and Cl suggests that these elements play a crucial role in modifying the surface structure through interactions.

Computational approaches have suggested that the most interesting feature of layered materials is their weak van der Waal forces between the layers, contributing to their lubricity. Also, several theoretical studies showed the relationship between the friction performance of layered materials and interlayer spacing.^11,29^ In this work, we develop a relationship between the interlayer spacing of ML-Ti_3_C_2_T_*z*_ MXene and its coefficient of friction.

The friction performance of the solid coating of ML-Ti_3_C_2_T_*z*_ MXene intercalated with different intercalating agents was investigated at a load of 1 N and a speed of 50 mm/s, as shown in Figure S10. Initially, all steel substrates used in this work were polished on a chemical mechanical polisher to ensure a uniform roughness of substrates. The duration of each friction test varied based on the specific parameters but generally lasted several minutes to ensure that steady-state conditions were reached. The COF was determined using eq 1:
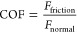
1where *F*_friction_ is the measured frictional force, and *F*_normal_ is the normal force applied during the test.

During our experiments, the normal force was applied by using a controlled loading mechanism to ensure consistent contact pressure between the surfaces. The frictional force was measured using a load cell or a similar device capable of detecting the resistance to motion between the surfaces. By dividing the measured frictional force by the applied normal force, we calculated the COF for each experiment. Multiple measurements were taken to ensure accuracy, and the average COF value was reported in the results.

**Figure 9:**
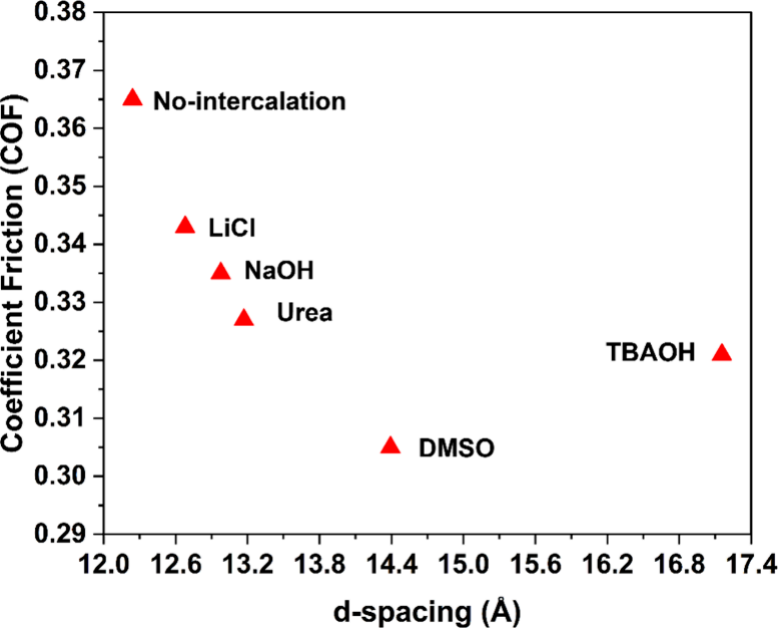
Relationship between the coefficient of friction and *d*-spacing of ML-Ti_3_C_2_T_*z*_ MXene coatings.

Figure 9 shows the relationship between the *d*-spacing of ML-MXenes and their coefficient of friction (COF). The average COF for each sample (Figure S12) was calculated after achieving the steady state conditions, which was after ∼250 cycles. The nonintercalated ML-Ti_3_C_2_T_*z*_ exhibited a COF value of 0.365. After the intercalation of ML-Ti_3_C_2_T_*z*_ the COF values drop to 0.305 (for DMSO-intercalated MXenes). Overall, the data confirm that the COF generally decreases as the *d*-spacing or interlayer spacing increases for multilayer MXenes. The quantitative analysis showed that the increase in interlayer spacing from 12.2 to 14.5 Å led to a reduction in the COF by approximately 20%. This relationship is supported by our experimental data, which are presented in Figure 1 and Figure 9. The decrease in the COF is attributed to the formation of a protective layer of sheared MXene nanosheets. Larger *d*-spacing allows for easier sliding of the layers over each other, resulting in lower friction.^11,12,20^ However, for TBAOH-intercalated ML-Ti_3_C_2_T_*z*_ MXenes, the COF value increases despite the increased *d*-spacing. We hypothesize that this may be due to the presence of residual TBA^+^. Residual TBA^+^ ions are relatively large organic cations and may introduce steric hindrance between the MXene layers, disrupting their smooth sliding. This steric effect can enhance interlayer interactions, leading to higher friction between the layers. Additionally, the presence of TBA^+^ ions could impede the shearing of layers under applied shear forces, further contributing to the observed increase in the coefficient of friction (COF). These findings are useful for selecting appropriate intercalation agents to control the friction properties of the ML-Ti_3_C_2_T_*z*_ MXene coatings.

The SEM images of the wear tracks for the solid lubricant coatings of intercalated ML-Ti_3_C_2_T_*z*_ MXenes, shown in Figure S13, highlight the influence of different intercalating agents on the tribological performance. The wear track in Figure S13a appears rougher than the other samples, with significant debris, indicating that the nonintercalated ML-Ti_3_C_2_T_*z*_ MXene provided less surface protection, resulting in a higher coefficient of friction (COF). In contrast, the wear tracks of intercalated MXenes (Figures S13b–f) show smoother surfaces with less prominent wear debris compared with Figure S13a. Notably, DMSO-intercalated ML-Ti_3_C_2_T_*z*_ MXenes displayed the smoothest wear track, suggesting the formation of a protective layer composed of single or few delaminated MXene layers on the steel substrate. Figure S13f, although smoother than Figure S12a, still shows notable debris accumulation, indicating partial surface protection. These findings confirm that the choice of intercalating agent is critical for optimizing the wear resistance of MXene coatings.

The correlation between electrical conductivity and the coefficient of friction for ML-Ti_3_C_2_Tz MXenes intercalated with different agents reveals (Figure S14) the interplay between electronic and tribological properties. As the electrical conductivity decreases (from no intercalation to intercalation with TBAOH or DMSO), the COF tends to decrease, suggesting that increased intercalation enhances lubrication but reduces the electrical performance. Essentially, the intercalation trades the balance between mechanical and electronic performance in an MXene-based lubricant by exchanging electrical conductivity for better friction performance.

## Conclusions

In conclusion, we controlled the interlayer spacing in ML-Ti_3_C_2_T_*z*_ MXene by chemical intercalation of various sizes of intercalating agents. The chemical intercalation of ML-Ti_3_C_2_T_*z*_ MXene with various intercalating agents led to an increase in interlayer spacing, which corresponded to the size of the inserted ions or molecules. The dispersion quality and colloidal stability of intercalated ML-Ti_3_C_2_T_*z*_ MXenes were evaluated, and no sedimentation was observed for 7 days. The increase in the *d*-spacing of ML-Ti_3_C_2_T_*z*_ MXene exhibited a decrease in the electrical conductivity and friction. Specifically, the enlarged interlayer gap led to reduced electrical conductivity in vacuum-filtered ML-Ti_3_C_2_T_*z*_ MXene films due to increased internal resistance. Furthermore, the increase of the *d*-spacing or interlayer gap in ML-Ti_3_C_2_T_*z*_ MXene resulted in a drop in the coefficient of friction. This reduction is attributed to the easy sliding of individual ML-Ti_3_C_2_T_*z*_ MXene layers under mechanical stress or load because the increased gap weakens the van der Waals forces.

## Supporting Information Available

Intercalating agents and their effective sizes, XRD and SEM of the MAX phase, optical microscopy of MXene dispersions, XPS of nonintercalated and intercalated ML-Ti_3_C_2_T_*z*_ MXenes, schematic of the sample preparation of solid lubricant coating, tribometer setup used for testing, SEM of a bare steel substrate, friction curve, SEM of wear tracks of lubricant coatings, and graph depicting the relationship between COF and electrical conductivity (PDF)
