# Supplementary material for: Effects of Intercalation on ML-Ti3C2Tz MXene Properties and Friction Performance
Source: ACS Appl Mater Interfaces. 2024 Nov 6;16(46):64156–65. doi: 10.1021/acsami.4c12659 (PMC11583124; doi:10.1021/acsami.4c12659)
Supplement: Supplementary file 1 — am4c12659_si_001.pdf [file am4c12659_si_001.pdf]

Supporting Information

The effects of intercalation on ML-$Ti_3C_2T_z$ MXene properties and friction performance

Kailash Arole[1], Savannah E. Pas[2], Ratul Mitra Thakur[2], Lara A. Amiouny[2], M Humaun Kabir[1], Milos Dujovic[1], Miladin Radovic[1], Jodie L. Lutkenhaus[1,2], Micah J. Green[1,2*], Hong Liang[1,3]*

[1]Department of Materials Science and Engineering, Texas A&M University,

College Station, TX 77843, USA

[2]Artie McFerrin Department of Chemical Engineering, Texas A&M University,

College Station, TX 77843, USA

[3] J. Mike Walker '66 Department of Mechanical Engineering, Texas A&M University,

College Station, TX 778843, USA

Corresponding author: micah.green@tamu.edu; hliang@tamu.edu

Table S1. The list of different types of intercalating agents used in this study and their effective sizes. **Note**: The species listed in brackets are the effective intercalants

Intercalating agents	Size of intercalants (Å)	Reference
**LiCl (Li^+)**	0.69	[1]
**NaOH (Na^+)**	1.02	[2]
**Urea (Urea)**	2.54	[3]
**DMSO (DMSO)**	2.93	[4]
**TBAOH (TBA^+)**	4.94	[5]

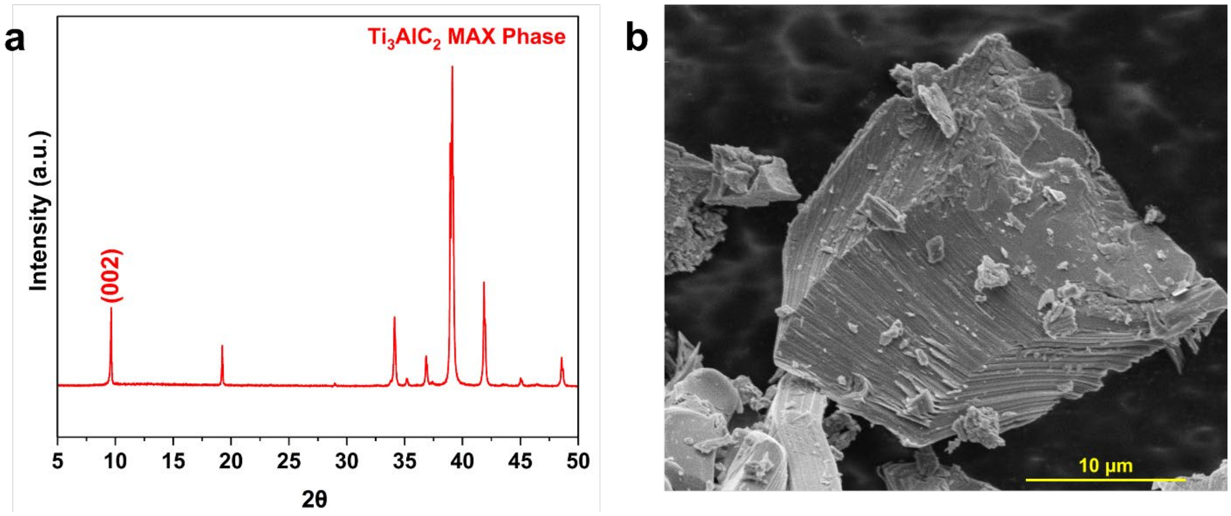

Figure S1. a) XRD and b) SEM of precursor Ti_3AlC_2 MAX used to synthesize the ML-$Ti_3C_2T_z$ MXenes.

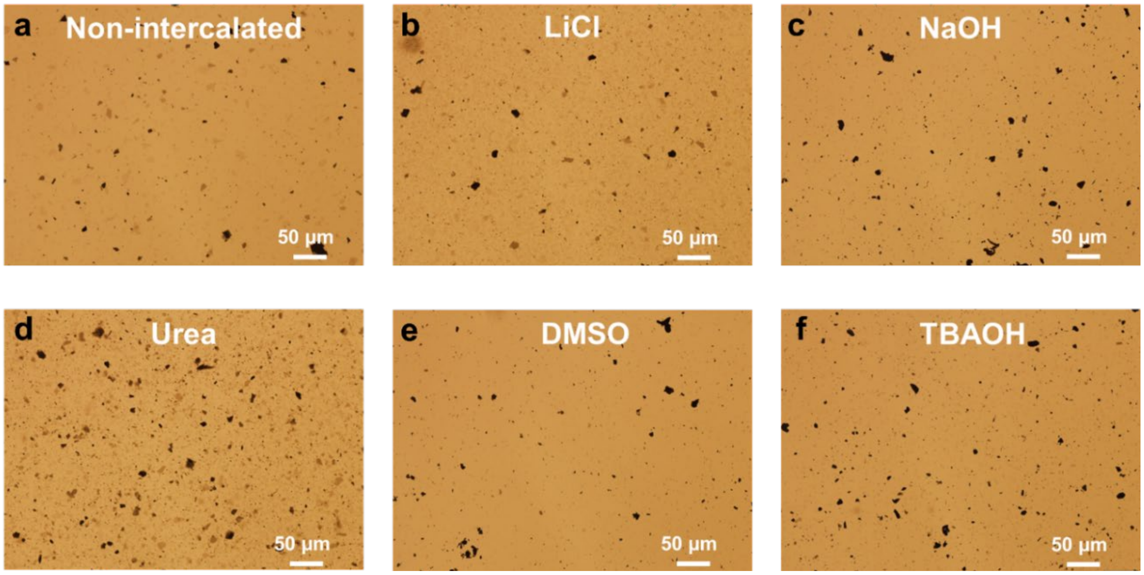

Figure S2. The optical microscopy images of intercalated ML-$Ti_3C_2T_Z$ MXene dispersions for (a-f) varying intercalants.

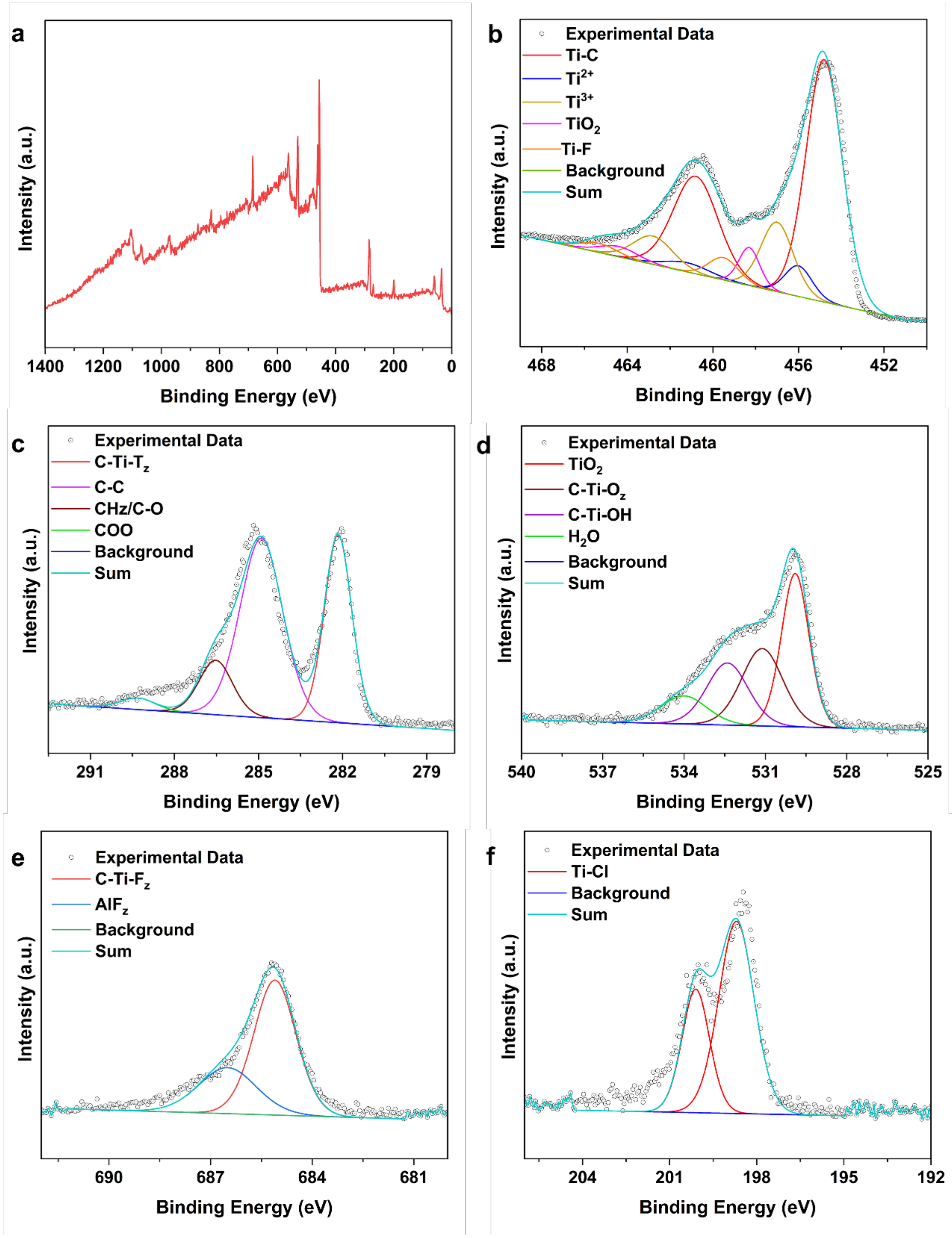

Figure S3. XPS spectra of non-intercalated ML-$Ti_3C_2T_z$ MXenes: a) survey; high resolution spectra of b) Ti 2p; c) C 1s; d) O 1s; e) F 1s; and f) Cl 2p

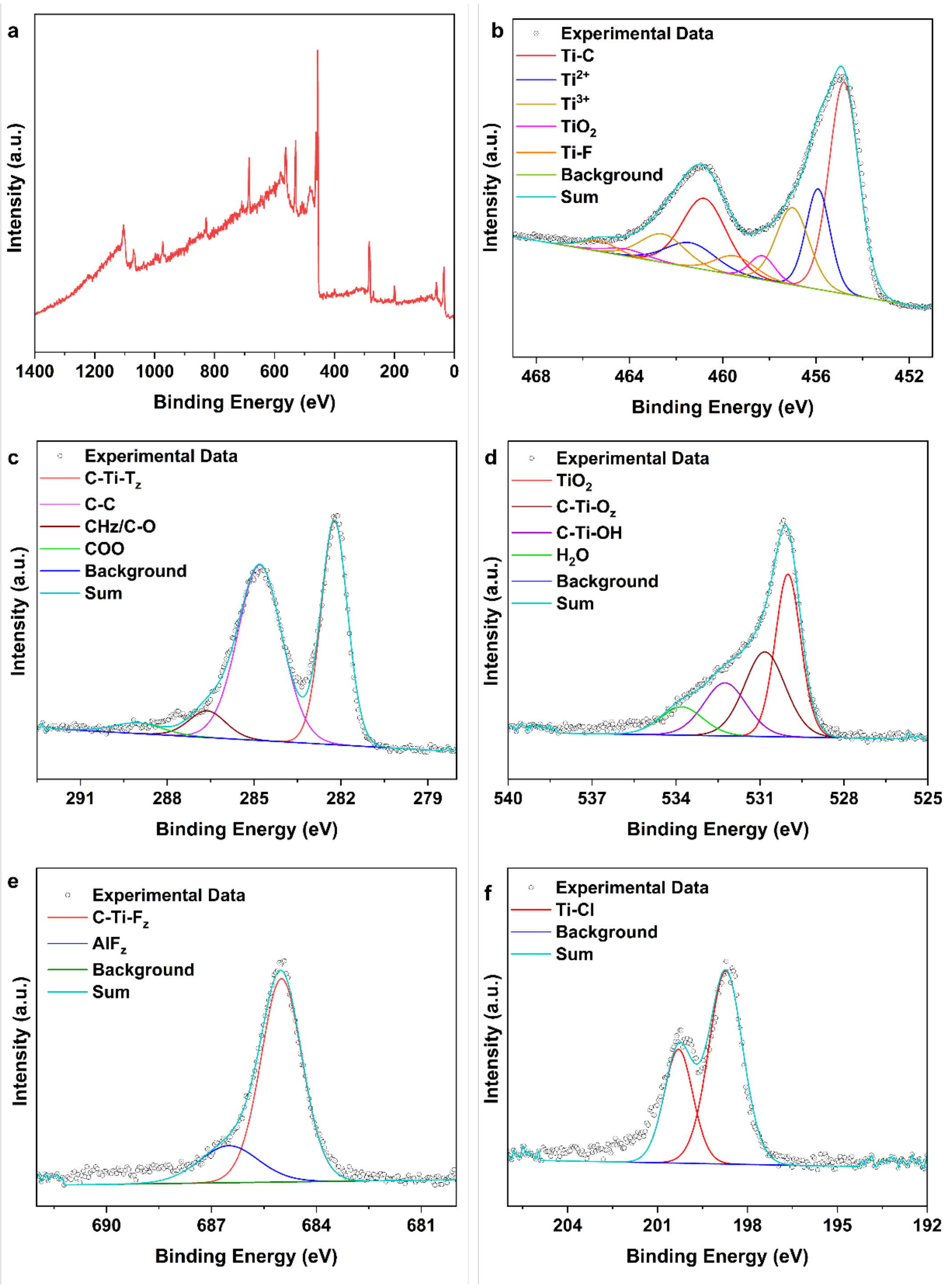

Figure S4 XPS spectra of LiCl-intercalated ML-$Ti_3C_2T_z$ MXenes: a) survey; high resolution spectra of b) Ti 2p; c) C 1s; d) O 1s; e) F 1s; and f) Cl 2p

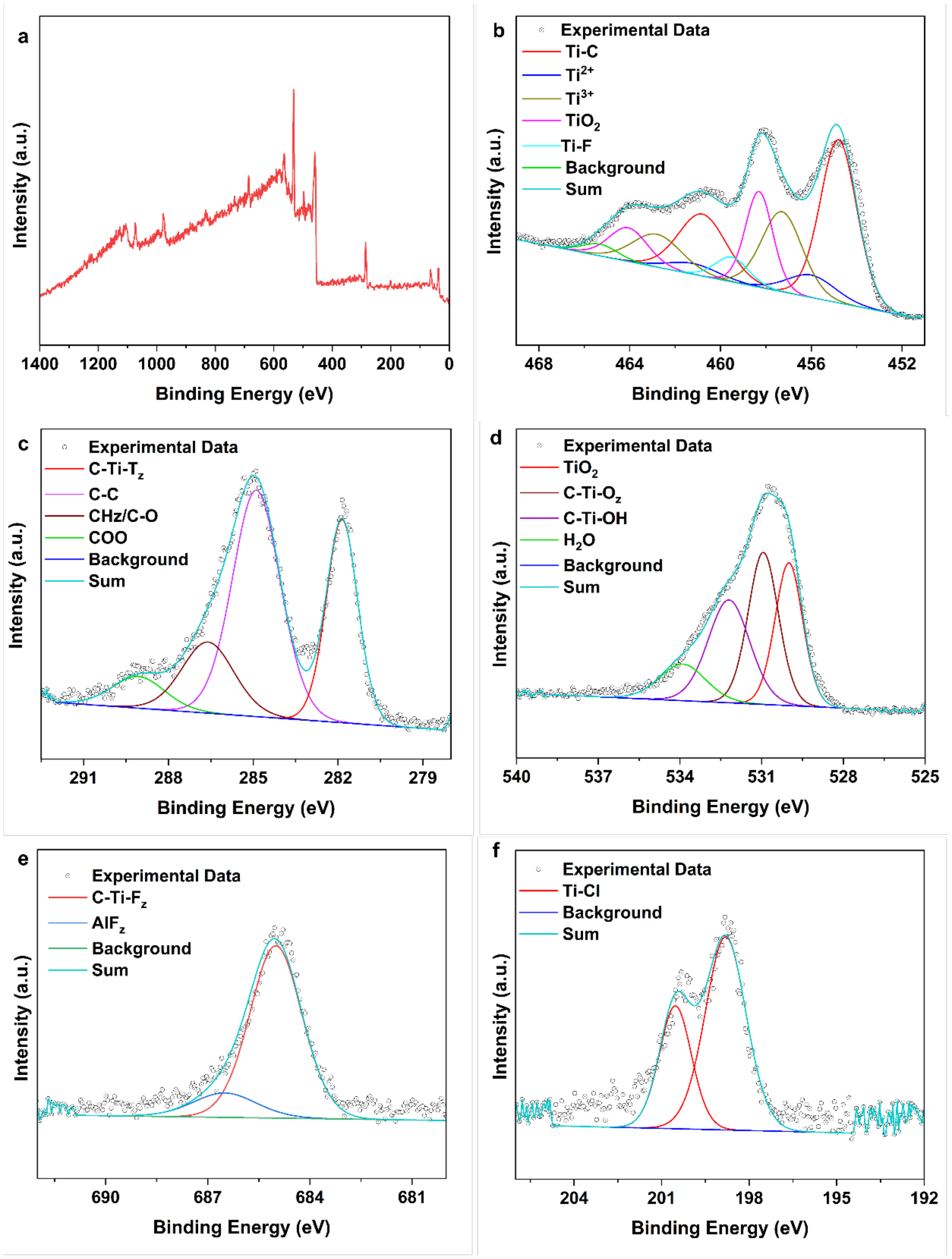

Figure S5. XPS spectra of NaOH-intercalated ML-$Ti_3C_2T_z$ MXenes: a) survey; high resolution spectra of b) Ti 2p; c) C 1s; d) O 1s; e) F 1s; and f) Cl 2p

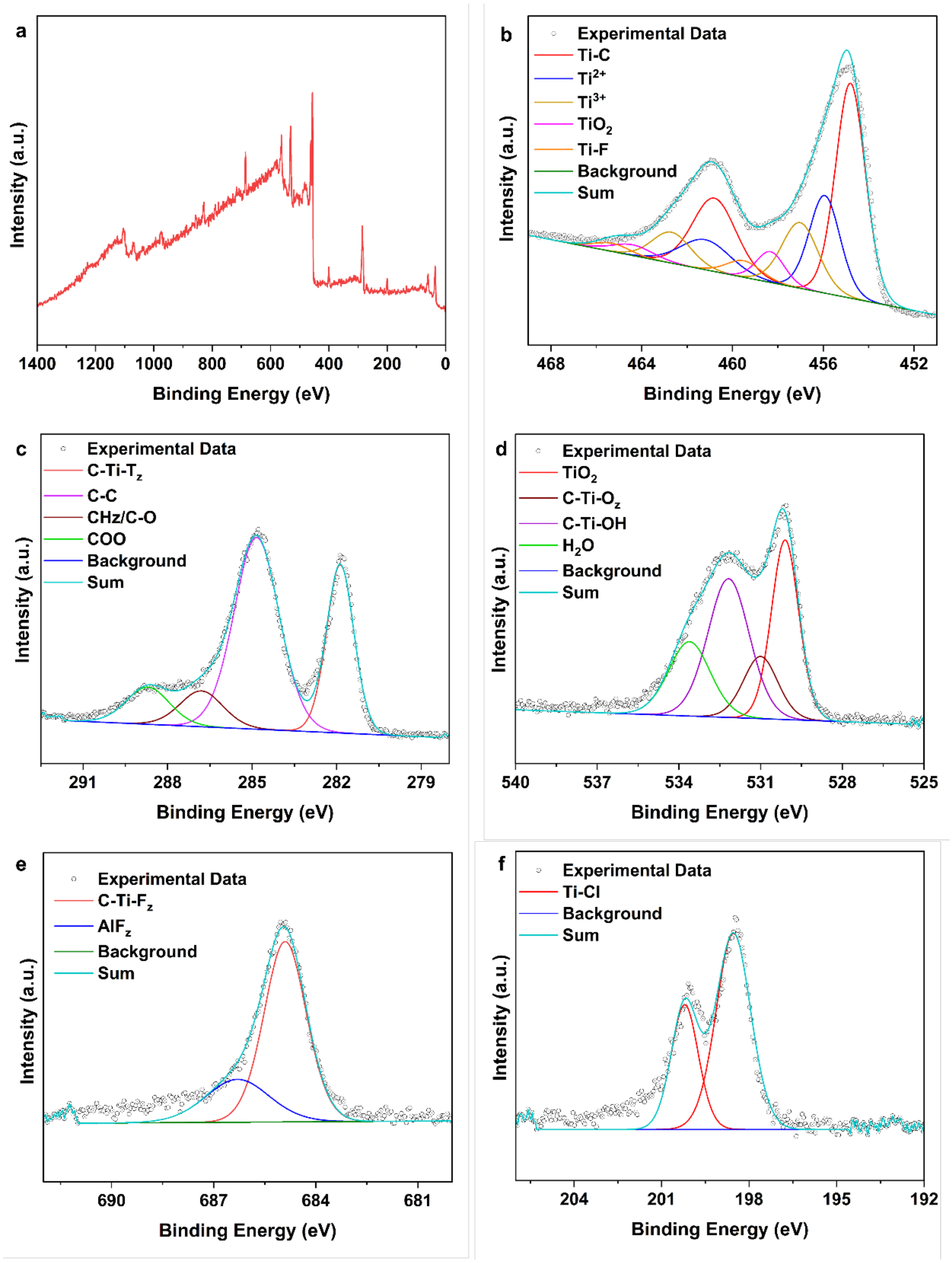

Figure S6. XPS spectra of Urea-intercalated ML-$Ti_3C_2T_z$ MXenes: a) survey; high resolution spectra of b) Ti 2p; c) C 1s; d) O 1s; e) F 1s; and f) Cl 2p

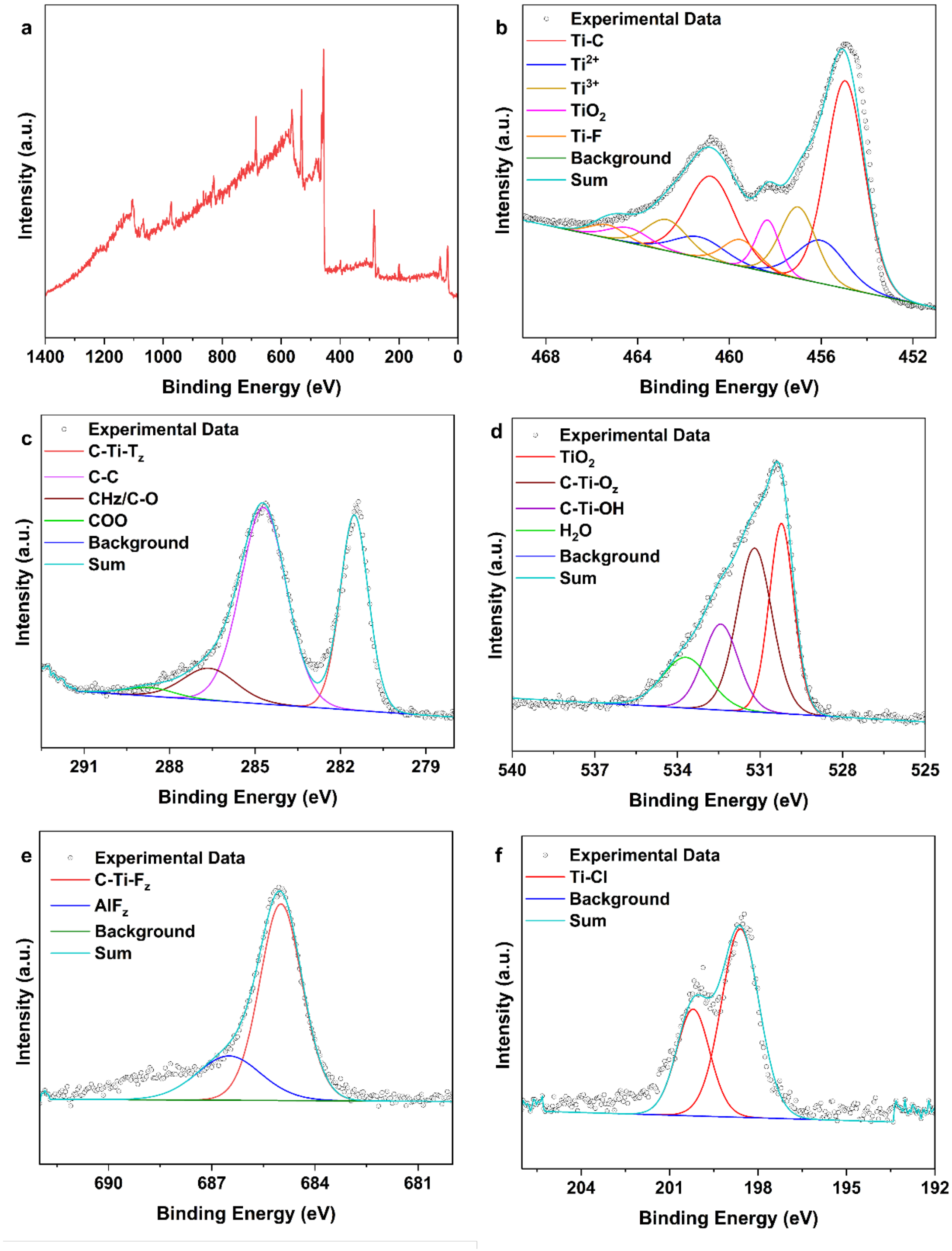

Figure S7. XPS spectra of DMSO-intercalated ML-$Ti_3C_2T_z$ MXenes: a) survey; high resolution spectra of b) Ti 2p; c) C 1s; d) O 1s; e) F 1s; and f) Cl 2p

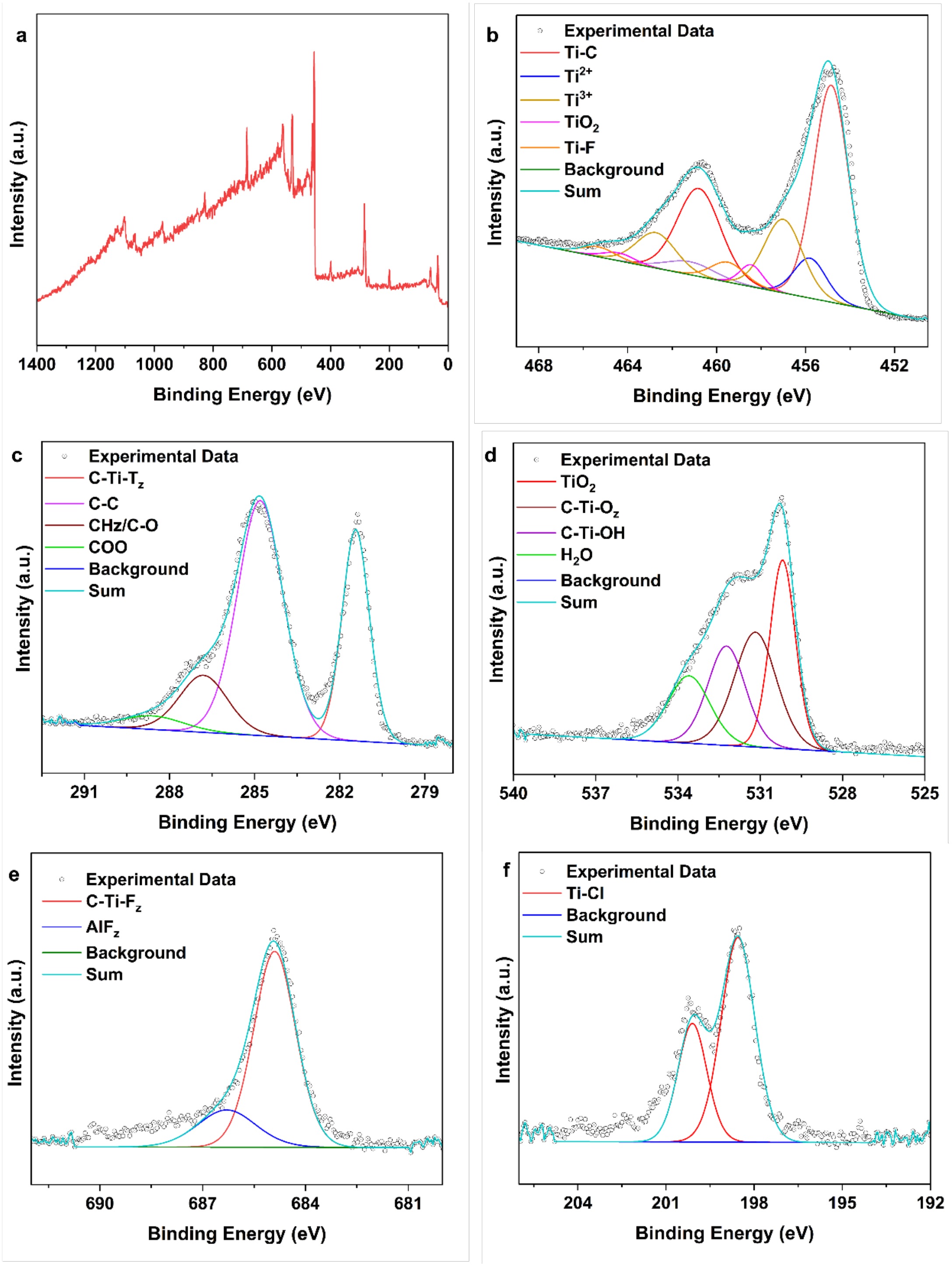

Figure S8. XPS spectra of TBAOH-intercalated ML-$Ti_3C_2T_z$ MXenes: a) survey; high resolution spectra of b) Ti 2p; c) C 1s; d) O 1s; e) F 1s; and f) Cl 2p

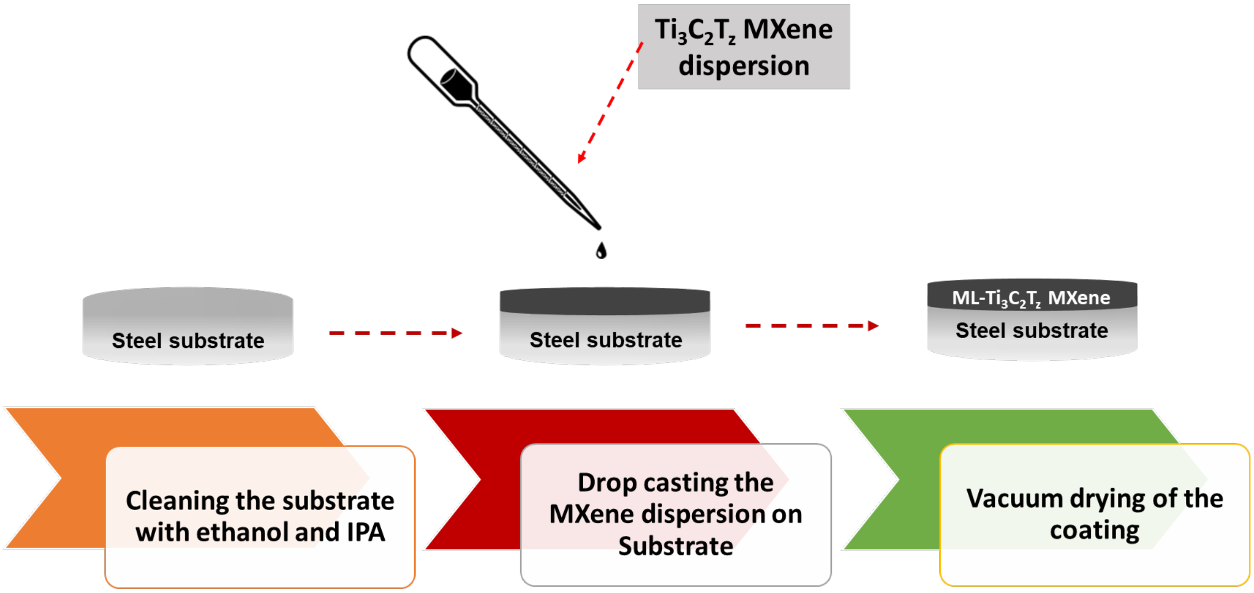

Figure S9. Schematic describing the solid lubricant samples preparation from ML-$Ti_3C_2T_Z$ MXenes dispersion.

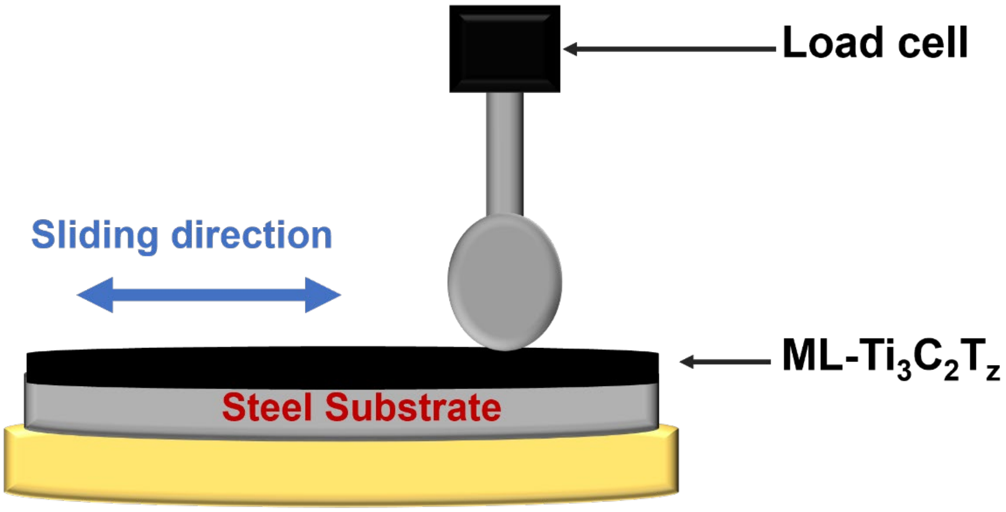

Figure S10. The setup used for the testing of ML-$Ti_3C_2T_Z$ MXenes solid lubricant coatings.

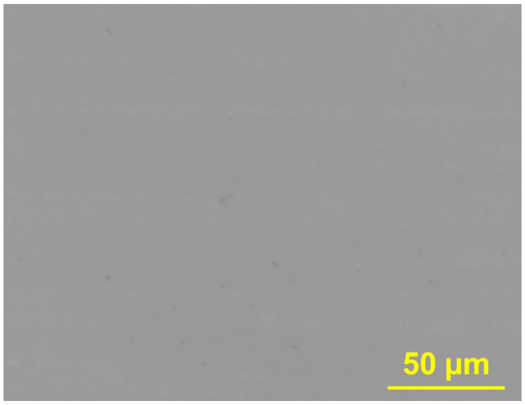

Figure S11. The SEM of the bare steel substrate used to coat the MXene dispersions.

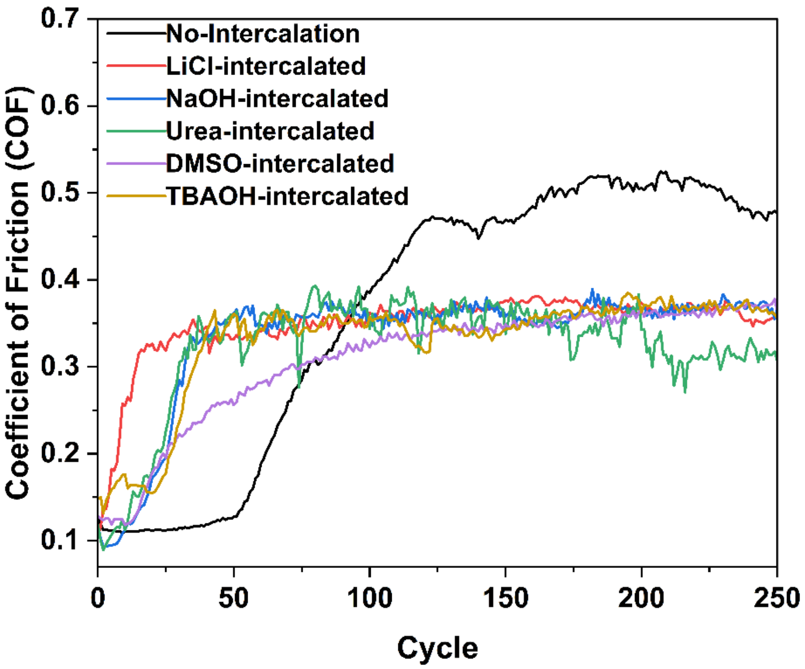

Figure S12. The friction curve of ML-$Ti_3C_2T_z$ MXenes solid lubricant coatings.

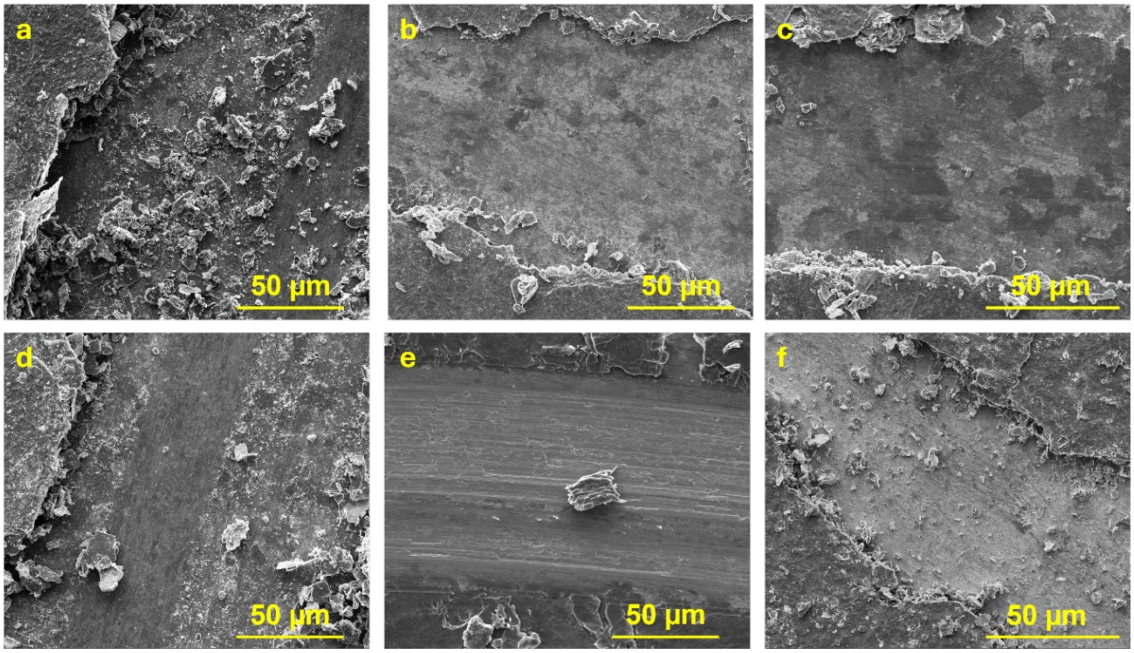

Figure S13. SEM of Surface morphology of wear track of ML-$Ti_3C_2T_Z$ MXene coated steel substrates intercalated with different intercalating agents: a) no intercalant; b) LiCl; c) NaOH; d) Urea; e) DMSO and f) TBAOH.

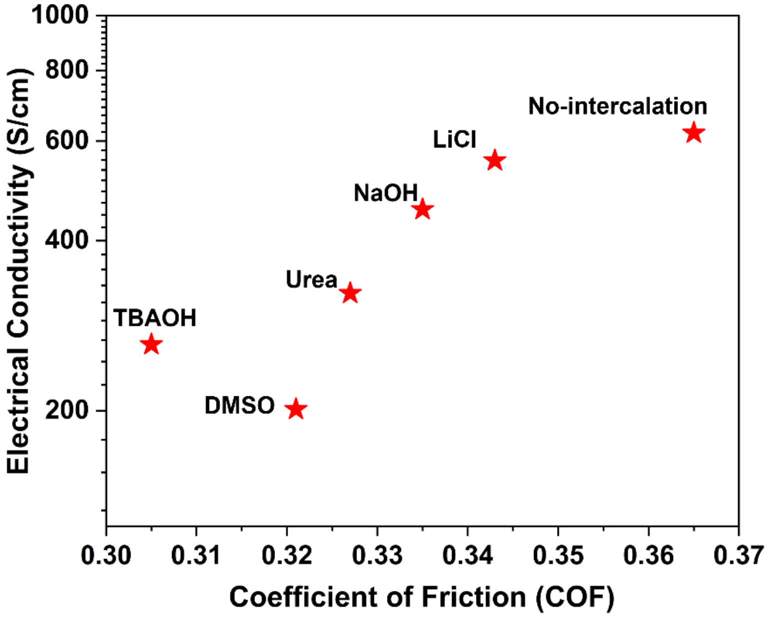

Figure S14. Correlation between electrical conductivity and coefficient of friction (COF) for ML-$Ti_3C_2T_z$ MXenes interclaated with different intercalating agents.

References:

(1) Long, Y.; Tao, Y.; Shang, T.; Yang, H.; Sun, Z.; Chen, W.; Yang, Q. H. Roles of Metal Ions in Mxene Synthesis, Processing and Applications: A Perspective. *Advanced Science* **2022**, *9* (12), 2200296.

(2) Natu, V.; Pai, R.; Wilson, O.; Gadasu, E.; Badr, H.; Karmakar, A.; Magenau, A. J.; Kalra, V.; Barsoum, M. W. Effect of Base/Nucleophile Treatment on Interlayer Ion Intercalation, Surface Terminations, and Osmotic Swelling of Ti3c2t Z Mxene Multilayers. *Chemistry of Materials* **2022**, *34* (2), 678-693.

(3) Sidey, V. On the Effective Ionic Radii for Ammonium. *Acta Crystallographica Section B: Structural Science, Crystal Engineering and Materials* **2016**, *72* (4), 626-633.

(4) Naguib, M.; Mashtalir, O.; Carle, J.; Presser, V.; Lu, J.; Hultman, L.; Gogotsi, Y.; Barsoum, M. W. Two-Dimensional Transition Metal Carbides. *ACS nano* **2012**, *6* (2), 1322-1331.

(5) de Larramendi, I. R.; Lozano, I.; Enterría, M.; Cid, R.; Echeverría, M.; Peña, S. R.; Carrasco, J.; Manzano, H.; Beobide, G.; Landa-Medrano, I. Unveiling the Role of Tetrabutylammonium and Cesium Bulky Cations in Enhancing Na-O2 Battery Performance. *Advanced Energy Materials* **2022**, *12* (2), 2102834.
